# Editorial: Immunomodulatory effect of nutrients on intestinal disorders and immunity

**DOI:** 10.3389/fimmu.2025.1552112

**Published:** 2025-02-04

**Authors:** Yi Wang, Ang Li, Yaozhong Hu

**Affiliations:** Tianjin Key Laboratory of Food Science and Health, School of Medicine, Nankai University, Tianjin, China

**Keywords:** nutrient, intestinal disorders, immunomodulation, immune aging, metabolism

Nutrition and immunity have emerged as active areas of research within the field of basic nutrition science, representing an interdisciplinary discipline that bridges nutrition and immunology. While traditional immunology focuses on the immune system’s defenses against exogenous substances, research on nutrition and immunity explores the relationship between dietary nutrients, dietary factors, nutritional status, and immune system function. Related research extends further, to examine the immune-mediated effects and interventions of nutrients on homeostasis and disease (1). Dietary nutritional components include macronutrients such as carbohydrates, fats, and proteins, micronutrients such as zinc (Zn) and iron (Fe), and dietary additives such as flavonoids and polyphenolic compounds. It's promising to conduct multidimensional studies to investigate the immunomodulatory functions and disease intervention effects of a variety of dietary nutritional components (2). It is imperative to elucidate the interactions between dietary components and the immune system, along with their intermediary roles in both health and disease. The identification of molecular mechanisms of nutritional immunoregulatory effects from a genetic perspective provides the foundation for multidisciplinary research, which aims to improve immune system function through nutritional interventions, ultimately promoting health and well-being.

The research on the exploration of the interactions between dietary components and gut microbiota is emphasized in the context of gut immunity, energy metabolism, and systemic immunity, which could systematically investigate the secretion and metabolic patterns of functional components like dietary functional factors, and nutrients, along with their health effects (3). For example, it is anticipated that research on infant immunity will be proposed to comprehensively examine the dynamic changes of nutrient human milk oligosaccharides (HMOs) and probiotics in breast milk during different stages of pregnancy, thereby elucidating their mechanisms in regulating gut immunity. Moreover, the bioactive mechanisms of dietary polyphenols on gut microbiota, intestinal homeostasis, and systemic inflammation are the intense field for dietary intervention.

The study is proposed to investigate the individual differences in metabolism and health effects of plant compounds. Focusing on energy metabolism and neurodegeneration, it is promising to evaluate the non-nutritional bioactivities and their efficacy in chronic disease interventions. The interaction of gut metabolism and immune function requires the systematic investigation of the interaction mechanisms and health effects of dietary functional factors, such as prebiotics and probiotics (4, 5). It is helpful to elucidate the immunoregulatory functions of dietary components and their role in maintaining systemic health homeostasis (6). By exploring the regulatory effects and mechanisms of dietary polyphenols and probiotics on innate and adaptive immunity, research is establishing health intervention effects based on the interactions between gut metabolism and the host system. Research is also clarifying the metabolic differences and application characteristics of specific metabolites. Studies are aimed at examining the immune regulatory functions of dietary components in the context of chronic diseases and provide a systematic analysis of their immune-enhancing effects, offering a scientific basis and data support for dietary-based immune regulation and immunity-based precision interventions.

In light of the increasing concern about aging and aging related dysbiosis, it is crucial to focus on nutrient mediated intervention for healthy aging and the reversal of age-related diseases (7, 8). Moreover, immune aging drives the functional decline of multiple systems and organs, which is closely related to systemic inflammation, recurrent infections, and age-related diseases in elderly individuals. Thus, delaying it is essential to contribute to healthy aging (9). Studies have found that a diverse diet can help reverse immune aging. For instance, supplementation with 2’-fucosyllactose (2’-FL) can significantly ameliorate age-related osteoporosis, metabolic syndrome, and systemic oxidative stress (10). It is speculated that 2’-fucosyllactose targets the mechanism related to immune aging. In any case, it is both necessary and important to expand the fundamental research on immunology and to investigate the results of nutritional immunology to develop novel intervention strategies for health and disease by leveraging their immunomodulatory effect.
